# A Review on Chronic Pain in Rheumatoid Arthritis: A Focus on Activation of NR2B Subunit of N-Methyl-D-Aspartate Receptors

**DOI:** 10.21315/mjms2020.27.1.2

**Published:** 2020-02-27

**Authors:** Ain’ Sabreena Mohd Noh, Che Aishah Nazariah Ismail

**Affiliations:** Department of Physiology, School of Medical Sciences, Universiti Sains Malaysia, Kelantan, Malaysia

**Keywords:** rheumatoid arthritis, N-methyl-D-aspartate receptor, NR2B subunit of N-methyl-D-aspartate receptor, chronic pain, autoimmune disease

## Abstract

Chronic pain is a debilitating condition that occurs after tissue damage, which substantially affects the patient’s emotional state and physical activity. The chronic pain in rheumatoid arthritis (RA) is the result of various autoimmune-induced inflammatory reactions in the joints. Both types of peripheral and central pain processing can lead to sensitisation. Non-steroidal anti-inflammatory drugs (NSAIDs) and disease-modifying anti-rheumatic drugs (DMARDs) can result in potent anti-inflammatory effect. However, these drugs are not able to suppress the pain from RA for a prolonged period. For years, researchers have examined the role of the N-methyl-D-aspartic acid receptor 2B (NR2B) subunit of N-methyl-D-aspartate receptors (NMDAR) in chronic and neuropathic pain models. This NMDAR subtype can be found in at the peripheral and central nervous system and it represents an effective therapy for RA pain management. This review focuses on the NR2B subunit of NMDAR and the different pathways leading to its activation. Furthermore, specific attention is given to the possible involvement of NR2B subunit in the peripheral and central pathogenesis of RA.

## Introduction

### Rheumatoid Arthritis as a Chronic Pain Disease

Rheumatoid arthritis (RA) is an autoimmune disease caused by the inflammation process in the body. It can lead to pain, swelling, and joint stiffness. In the long term, patients may experience symmetrical disabilities in the hands, wrists, and knees bilaterally (1). According to the United States Health Interview Survey (2013–2015), the annual prevalence of doctor-diagnosed arthritis was estimated at 22.7%. Women had a higher prevalence (23.5%) compared to men (18.1%). The prevalence also increased with advancing age (2). RA is the third most common type of arthritis, after osteoarthritis and gout (1). Commonly, RA causes destructive damage to soft tissues, joints and spinal column. Furthermore, some RA patients may present with extra-articular symptoms at the eyes, mouth, lungs and heart. These symptoms may manifest in the form of keratitis, pulmonary granulomas (rheumatoid nodules), pericarditis/pleuritic and small-vessel vasculitis (3, 4). The exact cause of RA remains unclear. However, it is likely to be triggered of by the complex interplay between lifestyle, genetic and environmental factors. Common risk factors of RA included infection, lung exposure to smoke, silica dust, nano-sized silica or carbon-derived nanomaterial (3). Some experts suggested stochastic factors as a possible cause leading to RA, especially among patients who are tested positive for anti-citrullinated protein antibodies (ACPA) (3).

Pain is the primary complaint of many RA patients. The pain is usually described as chronic in nature but with flare-ups in between, leading to fatigue, psychological disturbances, and poor quality of life (5). These prolonged symptoms can cause allodynia and hyperalgesia that resemble neuropathic pain. In a clinical study, Leffler et al. (6) discovered that RA patients with more than five years of symptoms demonstrated generalised allodynia to pressure, heightened sensitivity to light touch and hyperalgesia to innocuous cold, especially on the thigh area. Similar to other chronic diseases, RA pain is characterised as a complex integration of sensory, affective and cognitive processes that involve various abnormal cellular mechanisms at both peripheral (e.g. joints) and central (spinal, supraspinal, and descending system) levels of the nervous system (7). Inflammation is postulated to be one of the causes of the pain flares in RA patients, but there could be other factors at play. Furthermore, the intensity of the inflammatory markers is poorly associated with the measures of inflammation (8). Many studies have reported that the pain from RA persisted even when the inflammation is under control (8–10).

Current pharmacological approaches for RA management are directed at the immune system to suppress the symptoms. However, the impact of the central nervous system (CNS) on pain flares is poorly researched upon, according to the Pain Management Task Force of the American College of Rheumatology (11). Non-steroidal anti-inflammatory drugs (NSAIDs) and disease-modifying anti-rheumatic drugs (DMARDs) have a strong anti-inflammatory effect but they are not effective in improving RA pain (5). In a few clinical trials, the use of DMARDs, such as tumour necrosis factor-α (TNF-α) inhibitor, has been shown to effectively reduce RA pain during the early phase, but 40%–50% of the patients in a longer duration randomised clinical trial complained of unresolved pain at the end of the study (9, 10). In view of this, it is highly possible that RA pain is a result of CNS modification following the processing of pain signals (5). Moreover, it was also reported that the painful flares suffered by the RA patients were different from the inflammatory flares they experienced. In other words, the pain flares in RA may not be completely associated with significant joint swelling or increased erythrocyte sedimentation rate (12, 13).

Based on this postulation, it is crucial to discover the most effective analgesic to manage the prolonged pain in RA. To combat the pain derived from inflammatory arthritis such as RA, it is critical to explore the possible mechanisms leading to arthritic pain so that they can be modulated appropriately. In the past, many approaches have been suggested to find the best therapeutic option for arthritic pain. Targeting N-methyl-D-aspartate receptors (NMDAR) can be a promising option because they involved in the established pathways of chronic and neuropathic pain. This review focuses on the possible roles and mechanisms of NMDAR in the published arthritic and inflammatory-related researches. Specific attention is given to the role of the N-methyl-D-aspartic acid receptor 2B (NR2B) subtype on the pathogenesis of RA and how specific drugs can modulate its activation.

### NMDA Receptors and Related Subunits: Definition and Functional Properties

NMDAR is one of the ionotropic glutamate receptors (iGluRs). It is comprised of DL-α-amino-3-hydroxy-5-methylisoxasole-4-propionate (AMPA) and kainate receptors (14). As with AMPA receptors, NMDAR does not have specific selection for the cations that are permeable across its membrane (15). NMDAR has been found to be a key player in the transmission of excitatory inputs from primary sensory neurons to the brain via the spinal cord. The activation of NMDAR strongly contributes to the development of central sensitisation during chronic inflammatory pain and neuropathic pain. NMDAR can be found in the soma of primary afferent neurons in the dorsal root ganglia (DRG) (16–18), peripheral nerves terminals of primary afferent fibres in skin and muscle (19, 20) and trigeminal ganglion neurons (18, 19). Furthermore, it is also broadly distributed at the level of lamina I–II in the spinal cord (21) and several brain regions involved in the ascending and descending systems (21–25).

Similar to other iGluRs, NMDAR is a heterodimer made up of four subunits to form the ion channel collectively. N-methyl-D-aspartate receptor (NR)1 (contains eight splice variants), NR2 (A–D) and NR3 (A and B) are the basic subunits of NMDAR. NR1 is the fundamental channel-forming subunit of NMDAR (14, 24, 26, 27). However, for NMDAR to be functional, NR1 needs to combine with other subunits (at least one NR2 subunit). NMDAR can be formed as NR1/NR2/NR3 which displays reduced conductance and calcium permeability. It can also be formed as NR1/NR3 which only responds to glycine or D-serine regardless of the presence of glutamate (24). Other subtypes include a mixture of NR1/NR2A (termed as NR2A subunit of NMDAR), NR1/NR2B (termed as NR2B subunit of NMDAR) and NR1/NR2A/NR2B (28). These subunits of NMDAR are found on the synaptic and extra-synaptic sites of the neurons (26).

NMDAR activation is critical for the normal regulation of CNS. Nevertheless, any aberrant activation may cause neuronal excitotoxicity and subsequently lead to neurodegenerative disorders and chronic pain. NMDAR activation promotes either cell death or cell survival depending on the Ca^2+^ concentration and its route of entrance. It also depends on the type of NDMAR subunit structure and the location at which NMDAR is activated. Among the four subunits of NMDAR, the phosphorylation of NR1 and NR2 subtypes at C-terminals can modulate NMDAR activity and influence synaptic plasticity (3). Specifically, the activation of the NR2 subunit leads to neuroprotection or neuronal excitotoxicity. Two models have been developed to investigate these neuronal effects, namely the localisation model and subunit composition model. Both models show that activation of extra-synaptic NMDAR led to neurotoxic effects. On the other hand, the activation of synaptic NMDAR (i.e. NR2A subunit) shows a neuroprotective effect (29, 30). Compared to NR2A, the NR2B subunit has been more extensively studied. NR2B subunit is localised at extra-synaptic sites (postsynaptic density) (30), especially in the cerebral cortex, hippocampus and spinal cord regions (31). An increased activation of NR2B subunit results in a disproportionate amount of Ca^2+^ influx and overload in mitochondria, subsequently leading to excitotoxicity (16, 23). Ultimately, the downstream signalling cascade from N2RB activation promotes dendritic/synaptic damage, cell necrosis or apoptosis via a series of pro-death transcriptional responses (23, 30, 32).

### NR2B Subunit of NMDAR and Mechanisms Leading to Its Activation

The NR2B subunit of NMDAR is the main tyrosine-phosphorylated protein in the postsynaptic membrane (32, 33). The NR2 subunit is distinctive from other glutamate receptors as it possesses large intracellular C-terminal tails (approximately 630–650 amino acids for NR2A and NR2B subunits, respectively). Each tail comprised 25 tyrosine residues (33). Among the important tyrosine residues that are phosphorylated by serine family kinases (SFKs) are Y1252, Y1336 and Y1472 (33). Specifically, Y1472 is phosphorylated in the brain (34). The phosphorylation results in the recruitment of downstream signalling proteins. Sarantis et al. (35) revealed that the SFKs phosphorylation at Y1472 may interfere the relationship between NR2B subunit and clathrin adaptor protein 2. The interference can cause inhibition of receptor endocytosis, thus amplifying the synaptic NR2B subunit at NMDAR level.

During the induction of chronic pain, the NR2B subunit would be activated by the presynaptically-released glutamate. The activation produces depolarisation and excitatory postsynaptic potentials (EPSPs). When the depolarisation of postsynaptic membrane and the generation of EPSPs via spatial and temporal summation reach the threshold level, magnesium ion (Mg^2+^) would be dissociated from the NR2B subunit. These mechanisms lead to continuous development of EPSPs (15). The prolonged high-frequency tetanic stimulation ensures a repeated occurrence of synaptic plasticity. The prolonged synaptic plasticity is required for a long-term modification of excitatory synaptic strength so that activation and autophosphorylation of Ca^2+^/calmodulin dependent protein kinase II (CamKII) can take place. There are two ways this process can happen. Firstly, via protein kinase A (PKA)-induced activation of transcription factor cyclic adenosine monophosphate (cAMP) response element-binding protein (CREB). Secondly, via NMDAR-dependent activation of mitogen-activated protein kinase (MAPK) signalling cascades. Both activation pathways ensure the long-term potentiation (LTP) (15). A prolonged LTP serves as a positive feedback mechanism to drive a higher influx of Na^+^ and Ca^2+^ into the nerve via the high-frequency tetanic induction can cause. In turn, this results in further depolarisation to stimulate SFKs phosphorylation to form the NR2B subunit (23).

**Figure 1:**
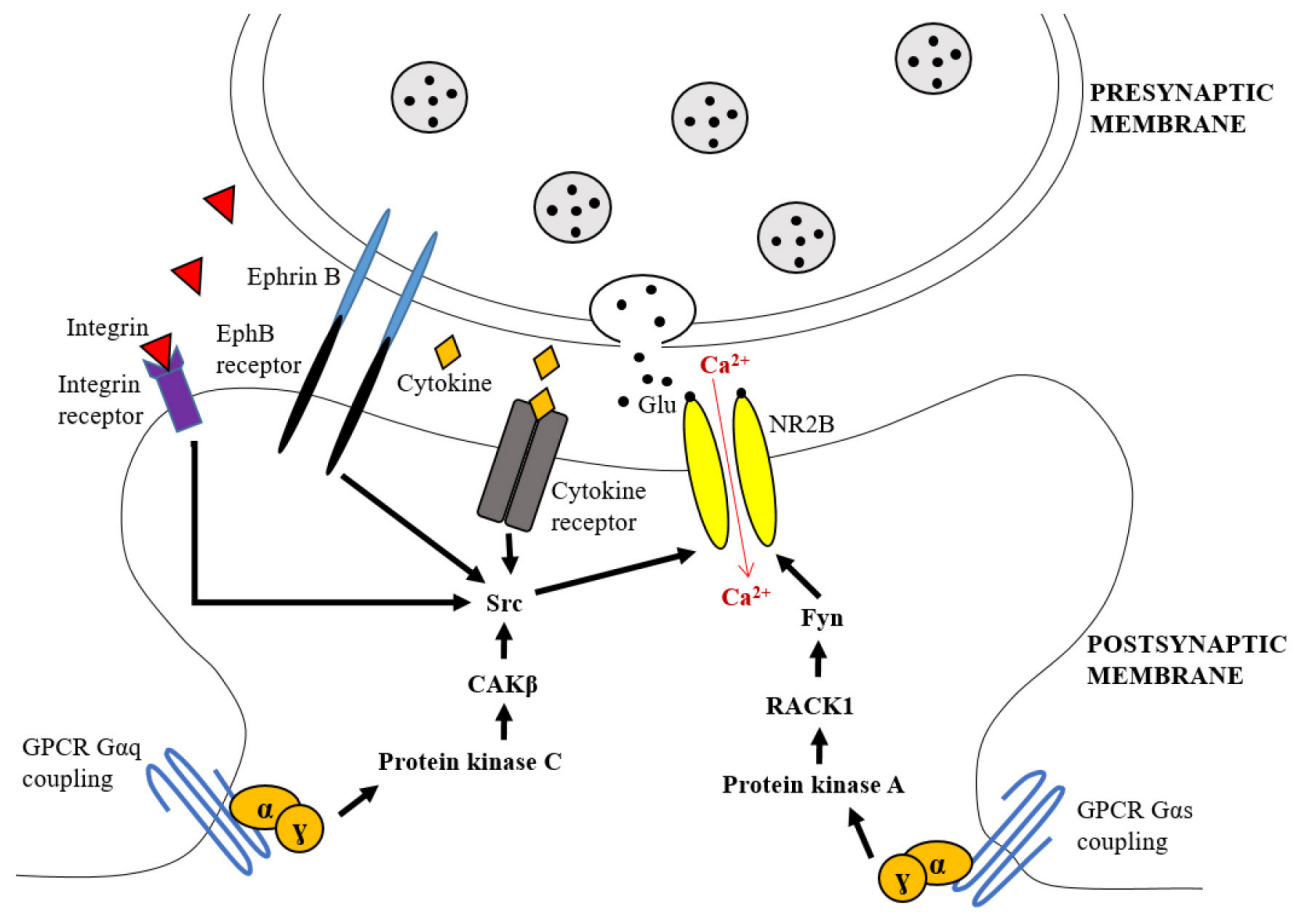
The cascades leading to the upregulation of NR2B subunit of NMDAR activity through activation of GPCRs, cytokine, integrin and EphB receptors. Gαq coupled-GPCRs transmit the signals via PKC and CAKβ to stimulate Src phosphorylation and causes the increased NR2B subunit activity. The Gαs-coupled GPCRs cascade through the activation of PKA leads to the dissociation of Fyn kinase from RACK1 to remove the suppression of Fyn and allowing it to upregulate the tyrosine phosphorylation of NR2B subunit leading to its elevated activity. Integrin binds to its receptor and activates Src phosphorylation on C-terminal of NR2B subunit via the activation of CAKβ. The EphB interacts directly with NR2B subunit by binding to EphB receptor at the postsynaptic membrane, causing the phosphorylation of this subunit Notes: RACK1 = receptor for activated C kinase 1; adapted from Salter and Kalia (33)

Furthermore, the stimulation of G-coupled-protein receptors (GPCRs) also induces the activation of the NR2B subunit that ultimately produces CNS plasticity. To elaborate, GPCRs are the sites where glutamate, acetylcholine, and dopamine neurotransmitters unite via their signalling pathways. The union of these neurotransmitters is necessary to change the gating or trafficking of NMDAR, including NR2B subtype. GPCRs include muscarinic, lysophosphatidic acid (LPA), and metabolic glutamate receptor 5 (mGluR5) receptors (24). These receptors may either amplify neuronal excitability or mediate the suppressive effects that diminish the neuronal excitability (21). There are several pathways by which GPCRs can activate the NR2B subunit. Firstly, GPCRs can directly bind to the NR2B subunit and change its activity or trafficking. Secondly, GPCRs can stimulate the cascade of second messengers connected to serine-threonine kinases such as PKA, protein kinase C (PKC) or Ca2+/ calmodulin dependent protein kinase (CamK). The stimulation of these second messengers results in an elevated phosphorylation of NR2B subunit. Lastly, GPCRs also send signals to receptor tyrosine kinase (RTK) such as epidermal growth factor receptors and platelet-derived growth factor receptors to regulate the activity of the NR2B subunit (Figure 1).

### Activation of NR2B Subunit of NMDAR via Gαq Coupling GPCRs Cascade

**Figure 2:**
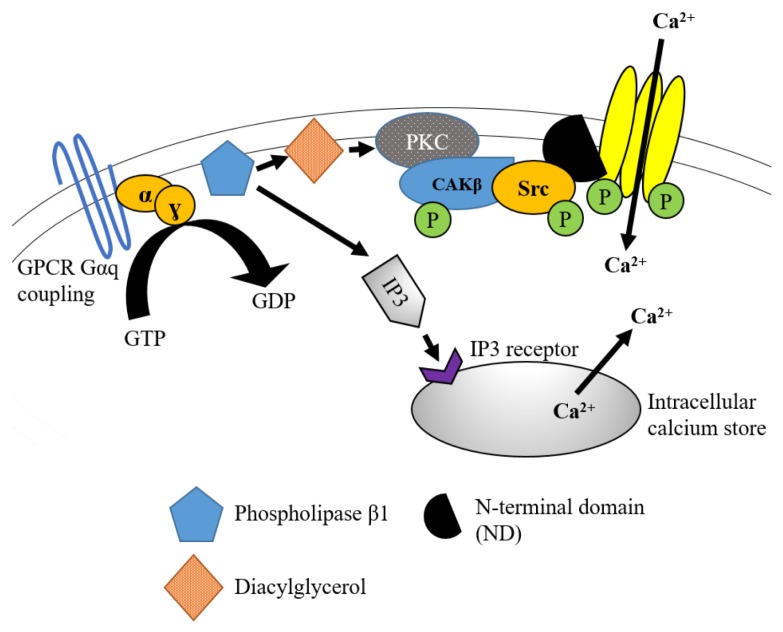
GPCR activation via Gαq coupling cascade leading to the increased activity of NR2B subunit of NMDAR. The activation of GPCR such as muscarinic, lysophosphatidic acid (LPA), metabolic glutamate receptor 5 (mGluR5) receptors promotes phospholipase activation and brings to the hydrolysis of PIP2 into IP3 and DAG. The binding of IP3 to IP3 receptor leads to the release of intracellular Ca2+ into the neuronal cytoplasm. The DAG stimulates PKC that altogether with the increased intracellular Ca2+ concentration promotes the stimulation of CAKβ which provides SH2 docking region for Src kinase to be activated. In turn, Src kinase leads to the upregulation of NR2B subunit activity via the binding to scaffolding protein N-terminal domain of this NMDAR subunit Note: Adapted from MacDonald et al. (15)

Specifically, when GPCRs via Gαq-coupling is stimulated, it promotes the activation of phospholipase (PLC). Activated PLC hydrolyses phosphatitylinositol 1,4, bisphosphonate (PIP_2_) into solubilised inositol-1,4,5-triphosphate (IP_3_) and diacylglycerol (DAG). IP_3_ then binds to the IP_3_ receptors at the endoplasmic reticulum membrane to induce the release of intracellular Ca^2+^ from the neuronal calcium store. On the other hand, DAG triggers the activation of PKC via the subtype PKCɣ. This subtype is primarily involved in nociceptive processing (36). Collectively, the elevated Ca^2+^ and activated PKC contribute to the activation of non-neuronal tyrosine kinase cell adhesion kinase (CAKβ) that would be auto-phosphorylated on its Y402 site (33, 37). The activation of CAKβ provides a high-affinity SH2 ligand for Src kinase. This ligand stimulates Src kinase phosphorylation at C-terminus of the NR2B subunit. Furthermore, CAKβ also acts by inhibiting the intramolecular networks that cause low-activity condition of Src kinase to ensure that activation can take place (21, 33). Eventually, this pathway leads to the upregulation of the NR2B subunit of NMDAR, as shown in a rat model of inflammatory hyperalgesia (3). The detailed mechanism is shown in Figure 2.

Although CAKβ plays a critical role in the activation of the NR2B subunit, it does not amplify the NR2B subunit currents on its own. In contrast, activated CAKβ stimulates the Src kinase activation via the production of LTP. Following that, the prolonged stimulation leads to further activation of CAKβ that can stimulate the activation of Src kinases. Simultaneously, this process hinders striatal-enriched tyrosine phosphatase (STEP) from inhibiting the NR2B subunit activity. Moreover, this mechanism may be further augmented as Src kinase also increases the intracellular Na^+^ level in the neuron (33).

### Activation of NR2B Subunit of NMDAR via Gαs Coupling GPCRs Cascade

**Figure 3:**
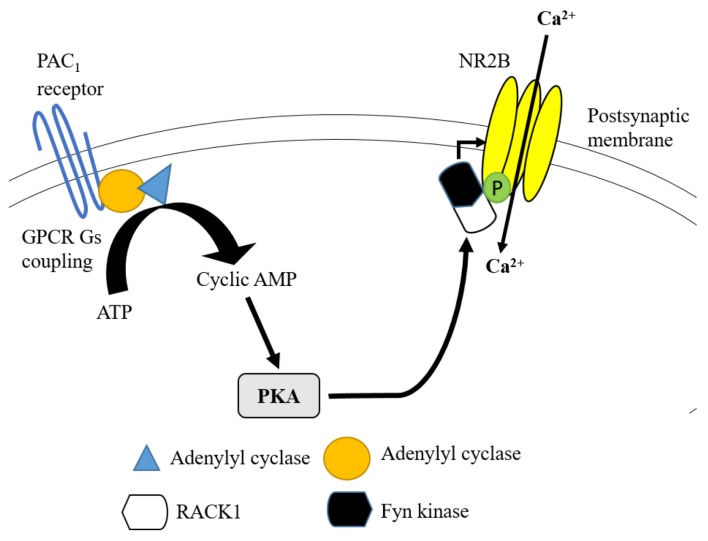
lternative GPCR via Gαs coupling cascade leading to the increased activity of NR2B subunit of NMDAR. GPCR activation via PAC1 receptor promotes adenylyl cyclase to be activated and converts ATP to cyclic AMP. This mechanism leads to the activation of PKA that allows the disintegration of Fyn kinase from RACK1. The free form of Fyn kinase acts on NR2B subunit to enhance its tyrosine phosphorylation and receptor activity Note: Based on ATP MacDonald et al. (15)

The cascade of Gαs-coupled GPCRs activation is another pathway that enhances the activity of the NR2B subunit during the inflammatory-induced pain transmission. This mechanism occurs through the binding of pituitary adenylate cyclase-activating peptide (PACAP) to PACAP type 1 (PAC1) receptors that are extensively expressed in the hippocampal region of the brain. PACAP regulates NR2B subunit activation in two ways. Firstly, the activated Gαq-coupled GPCRs pathway can further activate phospholipase C and PKC. The alternative way is via the activation of Gαs-coupled GPCRs pathway by binding to the enzyme adenylyl cyclase. Adenylyl cyclase able to produce cyclic adenosine monophosphate (cyclic AMP) from adenosine triphosphate (ATP). Cyclic AMP is needed to activate PKA. Following the stimulation of PKA, Fyn kinase and NR2B subunit are detached from the first WD propeller site of receptor for activated C kinase 1 (RACK1), resulting in the upregulation of tyrosine phosphorylation and activity of NR2B subunit (21, 33) (Figure 3).

### Activation of NR2B Subunit of NMDAR via Receptor Protein Tyrosine Kinase Pathway

The protein tyrosine kinase Ephrin B (EphB) is needed to increase the activity of the NR2B subunit. It tightly regulates the localisation and function of NMDAR at the synapse via its binding to EphB receptor on the postsynaptic density (33). This regulation takes place before the NMDAR undergoes posttranslational alterations via ɣ-secretase activity (38). In an in-vitro study, Ephrin B2 in the mature neuron was found to minimise the transient decline of NR2B subunit-mediated currents that produced the prolonged currents and elevated entry of Ca^2+^ into the neuron (38). Ephrin B also plays a role in the tyrosine phosphorylation of Y1252, Y1336 and Y1472 at the C-terminal tail of the NR2B subunit, all of which are SFKs phosphorylation regions. Ephrin B modulates these regions to induce the phosphorylation of the NR2B subunit. In an in-vitro study using HEK293 cells, both dominant-negative mutant of SFK and NR2B expression with SFKs-poor binding Ephrin B mutant demonstrated suppression of NR2B subunit tyrosine phosphorylation. As a result, the Ephrin B2-induced elevation of NR2B-subunit dependent intracellular Ca^2+^ level did not take place (34). In another preclinical study using Ephrin B knockout mice, the occurrence of LTP was reduced (39), thus highlighting the critical role of Ephrin B in producing synaptic plasticity. Collectively, these study findings emphasised that the signalling pathways which involve SFKs activation via Ephrin B activation were able to increase tyrosine phosphorylation and activity of the NR2B subunit.

### Activation of NR2B Subunit of NMDAR via Ras/MAPK/ERK Pathway

The MAPK is a family of serine/threonine protein kinases. When stimulated, they transduce several extracellular signals into intracellular transcriptional and post-transcriptional responses. MAPK also includes extracellular signal-regulated kinase (ERK) which comprises p38MAPK and c-Jun N-terminal kinase (JNK). The initiation of the ERK/MAPK pathway in the activation series of kinases follows the below sequence: Ras à Raf à MEK à ERK/MAPK (40). Apart from playing a vital role in cell proliferation and differentiation, ERK/MAPK activation cascades are also associated with neuronal plasticity and LTP. In collagen-induced arthritis pain model, the upregulation of the spinal NR2B subunit of NMDAR is closely associated with the activation of ERK1/2. Furthermore, the administration of analgesic (tramadol) is linked with adequate suppression of peripheral nociceptive hypersensitivity in mice (41). It is postulated that following the NR2B subunit-activated Ca^2+^influx, specific guanine nucleotide exchange factors (GEFs) would stimulate the phosphorylation of ERK (pERK) (28). The pERK is then translocated from the neuronal cytoplasm into the nucleus. In the nucleus, the pERK stimulates the ribosomal kinase-2 and causes the phosphorylation of CREB (pCREB) on serine 133. The pCREB further modulates the binding of certain genes containing CRE to a CREB binding site. The binding leads to the production of nociceptive responses via the actions of c-fos, neurokinin/substance P receptor (NK1), brain-derived neurotrophic factor (BDNF), dynorphin, calcitonin gene-related peptide (CGRP), and cyclooxygenase-2 (COX-2) (40). These neuromodulators trigger more activation of the NR2B subunit, leading to CNS plasticity that is reflected in the broadening of spinal cord neuron receptive fields. The end result of this pathway is the production of hyperalgesia and allodynia.

### Activation of NR2B Subunit of NMDAR via Cytokine Receptor and Integrin Pathways

During RA-induced inflammation, many pro-inflammatory cytokines are produced at the terminal ends of primary afferent fibres. Cytokines communicate by binding to their specific receptors at the terminal ends of the nerve fibres, resulting in the signal transduction process that causes inflammation. Since cytokines are pleiotropic in nature, they may act on different target cells and influence the role of other cytokines. The cytokines may act synergistically or antagonistically to stimulate an excessive inflammatory response. Interleukin-1 (IL-1), tumour necrosis factor (TNF)-, IL-17 and IL-6 superfamily are the cytokines that play a pro-inflammatory role. However, some of the cytokines may be anti-nociceptive or playing both pro- and anti-inflammatory roles (41).

IL-1β and TNF-α can be found abundantly in the synovial fluids and systemic circulation of the RA patients. They are released from the macrophages and monocytes via TLR4 activation (42). The binding of IL-1β to interleukin-1 type 1 receptor (IL-1R1) activates Src kinases. The activated Src kinases upregulates its phosphorylation at Y1472 on the C-terminal of NR2B subunit. In the cultured neuron of epileptic rats, the activation of IL-1R/Toll-like receptor (TLR) signalling potentiates the Src kinase-catalysed NR2B subunit phosphorylation. Subsequently, this produces an enhanced neuronal excitability (43). As for TNF-α, it is cleaved by TNF-α converting enzyme (TACE) into a soluble form. The soluble form becomes a ligand for two TNF receptors with different roles, namely TNFR1 and TNFR2. The stimulation of TNFR2 confers a neuroprotective role and leads to increased neuronal survival (44). In contrast, TNFR1 contains intracellular ‘death domain’ and its activation produces cell apoptosis, leading to early cell death via caspase-8 activation (42), as exhibited in the hippocampal neuron (44). Therefore, the stimulation of TNFR1 by TNF-α results in higher level of activation and recruitment of other inflammatory cytokines. It also directly activates reactive oxygen species (ROS)- and reactive nitrogen species (RNS)-productive enzymes that produce oxidative stress and neurodegeneration.

Integrins are heterodimeric cell-adhesion receptors that assist in cell-extracellular membrane interaction and cell-to-cell interaction. The activation of integrins can influence the activation of NR2B subunit. In RA patients with inflammation of synovial cells, integrins are involved in the production of pro-inflammatory markers and increased cellular feedback mechanism via inflammation (4). The binding of ανβ3- and α5β1-type integrins to their receptors can activate the SFKs. This activation takes place following the CAKβ activation that provides the SH2 docking sites on the C-terminal of NR2B subunit (4, 21, 33). The pro-inflammatory effect of integrin is also mediated via its activation of other signalling proteins, including RACK1 and MAPK following the Ras pathway. As a result, integrins leads to an increases production of nitric oxide (NO), prostaglandins E_2_ (PGE_2_) and vascular endothelial growth factor (VEGF) (4).

### Activation of NR2B Subtype in Pathogenesis of RA

The pathogenesis of RA involves a cascade of inflammation and chronic pain. The activation of NMDAR on the peripheral terminals of the primary afferents and DRG happens either via the direct stimulation of mechanosensitive cation channels or the indirect stimulation of inflammatory cytokines. These actions result in the transduction of pain signals from nociceptors and mechanoreceptors. As a result, sensitisation can develop without any tissue damage or inflammation. This review will discuss in detail the possible mechanisms of the activation of the NR2B subunit that leads to peripheral and central sensitisation in RA.

### Peripheral Sensitisation During RA

Articular changes in RA which may induce or sensitise primary afferent nerves, leading to pain at the extremities. The synovium and capsule of the joints are heavily innervated by postganglionic sympathetic nerve fibres and peripheral afferents of the DRG. In these areas, there is a large number of primary Aα and Aß sensory neurons that are involved in mechanosensation, and Aδ- and C-fibers that participate in nociception.

In RA patients, the presence of ACPA is likely the main cause of autoimmune reactions in the joints (3). ACPA included peptides such as fibrin, vimentin, fibronectin, Epstein-Barr nuclear antigen I (EBNA-1), α-enolase, type II collagen and histone. As a result of the autoimmune reaction, peptidyl arginine deiminase (PAD) enzyme undergoes catalysis and produces autoantibodies against these peptides (3). As the citrullination of neoantigens may induce MHC class II-dependent T cells activation, a high level of ACPA would halt the immunological tolerance. This, in turn, aids the B cells to produce more ACPA (3, 45). α-enolase, one of the ACPA, augments p38 MAPK NF-kB activity and binds to Grp78 on the surface of monocyte/macrophage, leading to the release of TNF-α, IL-1α/ß, interferon-ɣ and PGE_2_ (45–47). The binding of inflammatory cytokines to the cytokine receptors expressed on the postsynaptic neurons (e.g. IL-1β to IL-1 receptor/Toll-like receptor superfamily) potentiates NR2B subunit activation. This process happens via the upregulation of the tyrosine phosphorylation on its c-terminal. Subsequently, there is an increased influx of Ca^2+^ into the neurons (33, 48). Apart from that, macrophages and monocytes also produce histamine, bradykinin, serotonin, ATP and protons (H^+^) following inflammatory responses. Substance P, CGRP and somatostatin are released from peripheral nociceptive fibres to further modulate inflammatory process. They also act by stimulating the auto activation of the sensory neurons through cognate receptors expressed on the nerve endings (49). Consequently, substance P induces plasma extravasation and increases immune cell recruitment, T cells production and mast cell degranulation. It also stimulates cytokine release from macrophages and induces the proliferation of fibroblasts and endothelial cells (50). These neuromodulators and inflammatory mediators sensitise the peripheral ends of Aδ- and C-fibres by binding to the receptors expressed on the nerve surface (47).

In the nervous system, several ion channels are involved in the detection of noxious and innocuous inputs. For example, transient receptor potential (TRP) channels such as TRPV1-4 and TRPM3 detect noxious heat sensation whereas TRPA1, TRPC5 and TRPM8 are associated with noxious cold temperatures. TRPA1 and TRPV4 subunits detect noxious mechanical signals while TRPA1, TRPV1, TRPV3, TRPM8 and TRPC3 are involved in the transmission of itch sensation (21). These ion channels are heavily expressed on Aδ- and C-fibres. The released inflammatory mediators and neuromodulators act on these selected receptors and ion channels to generate APs. For example, PGE_2_ stimulates the phosphorylation of the tetrodotoxin-resistant sodium channel to increase the excitability and reduce the threshold of nociceptor (51). On the other hand, H^+^ acts on the TRPV1 and TRPA1 mediated by Gαq-coupled B_2_ receptors in the DRG (21, 47). As for histamine, it acts on the TRPA1 channel mediated by Gαq-coupled H_1_ receptors and PKC activation (52). Nerve growth factor (NGF) sensitises TRPV1 by early stimulation of PI2 kinase and the presence of PKC and CamKII (53). These altered TRP channels, along with acid-sensing ion channels (ASICs), mechanosensitive K^+^ and Piezo channels, and voltage-gated Na^+^ and Ca^2+^ channels activate GPCRs (21). It is also suggested that the increased cytosolic Ca^2+^ from GPCRs activation does not produce sensitisation, but instead causes the immediate channel desensitisation during prolonged inflammatory reaction (21). Evidently, the phosphorylation of PKA-mediated TRPV1 channels enhances their sensitivity towards the inflammatory mediators. As a result, there is a decrease in the desensitisation mediated by Ca^2+^ (54).

DRG is the first relay neurons in the propagation of innocuous and nociceptive signals. The innervated peripheral nerves transform the noxious stimuli produced from the actions of inflammatory mediators (47) into APs to be propagated to the spinal cord (55, 56). During the sensitisation, the transformation into APs is facilitated either via reduced peripheral nerve threshold, increased supra-threshold responses or coordinated action of various ion channels mechanisms (21). The subsequent peripheral sensitisation is characterised by a high spontaneous activity and a reduced threshold of nociceptive fibres activation. It can also be a result of aberrant responsiveness with local production of neuropeptides following the nerve activation (8).

To date, the arthritic process involving NMDA receptors at the peripheral region has not been extensively studied. However, available research has detected the expression of NMDAR in the rat model of visceral pain, especially at DRG neurons (57). In a pre-clinical study, Ma and Hargreaves (22) discovered a profound expression of the NR2B subtype on the small and medium-sized diameter primary afferents. The NR2B subtype is also significantly expressed on the surfaces of satellite glial cells that encapsulate the DRG neurons (56). During the autoimmune responses, the increased cell-surface trafficking of DRG neurons with the binding of inflammatory and nociceptive mediators depolarises the membrane. During the nociceptive transmission, glutamates are released and they bind to the NMDARs, including the NR2B subtype. Besides that, the massive expression and stimulation of voltage-dependent Ca^2+^ channels also lead to increased permeability of these ion channels and Ca^2+^ influx into the neuron (58). N- and T-type channels are also increasingly upregulated during the occurrence of chronic pain (59). In short, these mechanisms are the formation of the spontaneous pathological activity and excessive spontaneous firings at the afferent neurons.

### Central Sensitisation During RA

In RA, there is an extensive central nociceptive processing involved in the development of RA pain. Therefore, the extent of tissue inflammation or damage at the peripheral articular joints is not the only predictive factor of the presence and severity of RA pain. During the pain transmission to CNS, any impaired or pathological state of CNS may contribute to the development of chronic pain. The central modification also influences the psychosocial factors of pain perception (8). Spinal cord dorsal horn is the first relay for pain propagation in CNS. Among all the NR2 subunits, the NR2B subunit of NMDAR is more prominently distributed and expressed in the superficial laminae (lamina I–II) of the spinal cord dorsal horn (20, 60). In the event of chronic inflammation, the sensitisation of inflammatory mediators on the low-threshold mechanosensitive Aβ-fibres may extend widely into laminae I and II of the spinal cord. This extension leads to the formation of synaptic networks with nociceptive-specific neurons, among which are the pathways that activates the NR2B subunit (61). As a result, there is an increased influx of Ca^2+^ into the neurons that subsequently stimulates the CamKII cascades that phosphorylate NR2B subunit and other receptor-ion channels (26, 62). The nociceptive signals received from laminae I and II of the spinal dorsal horn are transmitted in an ascending manner to various parts of the brain to be processed, including parabrachial nucleus and thalamus. From these regions, the signals are then transmitted to the somatosensory cortex, amygdala, prefrontal cortex, insular cortex and anterior cingulate cortex (ACC) to be processed as negative sensory and emotional experiences from the chronic pain (25). Clinically, the alterations in the cortical opioid receptor binding in these brain regions may account for the insomnia and depression experienced by RA patients (8).

Furthermore, during chronic inflammation, pro-inflammatory mediators such as IL-1β and TNF-α are systemically released by lipopolysaccharide/Toll-like receptor 4 (LPS/TLR4) binding-activated monocyte or macrophage. These mediators can across the blood-brain barrier to activate microglia in the spinal cord and brain during autoimmune-induced inflammation (42). These non-neuronal cells induce further release of pro-inflammatory markers and oxidative markers via its P2X7 receptor activation (63). This results in further sensitisation of neurons and microglia that continues to augment the activity of the NR2B subunit expressed on their cell surfaces. The activation of TNFR1 by TNF-α also triggers the release of glutamate from astrocytes via astrocytic GPCRs activation. This process also leads to increased activity of the NR2B subunit (64). Persistent activation of CNS-selective TNF expression may contribute to spontaneous chronic inflammation and also triggers the apoptotic pathways that leads to early cell death (44).

Several brain regions are reported to demonstrate a strong upregulation of the NR2B subunit of NMDAR during chronic inflammation. In a rat model of complete Freund’s adjuvant (CFA) induced-inflammation, the injection of NMDAR antagonist MK-801 prominently blocked the aberrant spontaneous discharges and pain-evoked discharges at the arcuate nucleus site. This finding is highly suggestive to be related to the increased phosphorylation of the NR2B subunit mediated by PKC and subsequently resulted in the alleviation of thermal and mechanical hyperalgesia (36).

Undeniably, the development of RA pain involves more than just the ascending nociceptive transmission pathways. It is also associated with an impaired descending inhibitory mechanism that may negatively facilitate the spinal transmission of pain. Under normal physiological conditions, the excitatory and inhibitory interneurons in the spinal dorsal horn lamina form a complex linkage that processes modality-specified somatosensory inputs in a proper manner. However, this system progressively collapses in the event of chronic pain, particularly in cases of developed mechanical allodynia. Moreover, the inhibitory glycinergic signals stimulated by Aβ-fibres to form a feed-forward inhibitory mechanism under the normal situation are also disrupted. As a result, the PKCɣ in lamina II of spinal dorsal horn that receives the mechanical and nociceptive signals from Aβ-fibres is activated (25). The activation of PKCɣ subsequently induces SFKs and contributes to the increased tyrosine phosphorylation of the NR2B subunit.

Apart from the spinal cord, other regions categorised under the descending circuit include brainstem ACC, periaqueductal gray (PAG), and rostral ventromedial medulla (RVM). These brain regions play a dual role. They can either inhibit or facilitate nociception, depending on which cells are activated. Some studies have proven that NMDAR expression on these regions plays a significant role during chronic pain, such as RA pain (25, 27). For example, during acute or chronic pain, ACC plays a bigger role in facilitating the nociception rather than initiating the inhibitory circuits (62, 65). The introduction of high-intensity electrical stimulation at most sites of the ACC did not trigger any anti-nociceptive effect. Although the descending stimulation from ACC relays at the RVM, it is found to be independent of RVM activity, leading to an exaggerated spinal sensory transmission (62). In a chronic inflammatory-induced pain model, the synaptic currents mediating NR2B subunit in the ACC region of the forebrain of the mice were significantly upregulated. Thus, there is a significant association between NR2B subunit activity and inflammatory-induced behavioural sensitisation (66). In addition, since ACC synapses have high plasticity (62, 65), they can contribute to the development of LTP. Following chronic inflammatory pain, there are two types of LTP in the ACC, namely postsynaptic (post-LTP) or presynaptic forms of LTP (25). The post-LTP is driven by the glutamatergic NR2B subunit via the stimulation of adenylyl cyclase type 1 (AC1) and PKA (67).

In another study, using CFA-induced chronic inflammatory pain model, Hu et al. (27) found a strong activity of the NR2B subunit of NMDAR that was selectively upregulated in the PAG. RVM is an intermediate in PAG to modulate pain as this region is a crucial integrated relay in the descending modulation of pain (27, 68, 69). The activation of ON cells in RVM results in the facilitation of nociceptive transmission while the stimulation of OFF cells mediates the inhibition of nociceptive processing (69). These dysfunctional roles of inhibitory pathways are directly associated with the occurrence of allodynia and hyperalgesia during chronic pain condition, as evidenced by two studies. In the first study, reduced mechanical allodynia was detected in the CFA-induced inflammatory rat model following the microinjection of NR2B subunit antagonist (66). In another study, dose-dependent facilitation of tail-flick reflex took place after the microinjection of NMDA into the RVM of the rat (70).

### Direct Inhibition of NR2B Subunit of NMDAR May Alleviate RA Pain

Although there is no specific cure for RA, there are several therapeutic strategies to expedite the diagnosis and to achieve a low disease activity state. Published research has shown that the application of several NMDAR antagonists can reverse the hyperalgesia and allodynia in several models of chronic pain to the normal or pre-inflammatory states. For example, MK-801 manages to reduce the mechanical hypersensitivity in a rat model of inflammation (71) while DL-2-amino-5-phosphonovaleric acid (AP-5) profoundly inhibited the C-fibre evoked responses of dorsal horn neurons (72) in animal models of nociception and inflammation. In the mice model of chronic arthritis, tramadol, another NMDAR antagonist managed to alleviate the peripheral pain hypersensitivity via the strong suppression of ERK2-signalling NR2B subunit activation (23). Furthermore, the inhibition of the NR2B subunit in the descending inhibitory circuit can also reduce the chronic symptoms related to RA. The introduction of NR2B subunit antagonist, Ro 25-6981 into the PAG region of the brain effectively alleviated thermal hyperalgesia of CFA-induced peripheral inflammatory pain in a rat model (27).

In addition, a previous investigation examined the effect of ifenprodil, a non-competitive NR2B subunit of NMDAR antagonist, on the pain response in a rat model. When given intrathecally for seven days, ifenprodil prominently inhibited the NR2B subunit expression in the spinal cord region of a painful diabetic neuropathy rat model, thus alleviating the tactile allodynia and formalin-induced pain responses. Furthermore, in the preclinical phase of another two studies, this drug was found to act synergistically with levodopa (L-DOPA), tricyclic antidepressants and selective serotonin reuptake inhibitors used to treat depression in Parkinson’s disease (73, 74). There is also evidence to suggest that CP-101, 606, a compound of ifenprodil (also known as the α_1_-adrenoceptor antagonist and selective NR2B subunit antagonist) can inhibit the activation of NR2B subunit via interaction on the polyamine modulatory site (75). More remarkably, the NR2B subunit of NMDAR antagonists has shown no adverse side effects in humans thus far. This finding is important because adverse effects from non-selective NMDAR antagonists have been reported even when they are consumed at neuroprotective doses (75, 76). In short, it is clear that the activation of the NR2B subunit plays an important role in nociceptive transmission. Therefore, the termination of NR2B subunit activation may be an effective therapy for the arthritic pain in RA patients.

## Conclusion

Despite the availability of certain biologics to suppress and control the inflammatory responses in RA, the pain resulted from RA warrants more attention. The reduction of inflammation does not mean the patients will be pain-free. For clinicians, pain may just influence disease assessment and treatment choices. However, for RA patients, pain may be their worst problem as it affects their emotions and working ability. Current medications are not potent enough to reduce arthritic pain. For effective management of RA, clinicians need to be more concerned about the patients’ pain symptoms to outline a better pain management plan. Therefore, the search for appropriate therapeutic approaches that can modulate the pain is critical to relieve the suffering of RA patients. The roles of the NR2B subunit of NMDAR especially in chronic and neuropathic pain management are gaining serious attention. Previous studies have shown that the antagonistic action of this N2RB may be effective in chronic pain management. However, more experimental studies and clinical trials need to be conducted to elucidate its possible roles in modulating RA pain. It is hoped that in the future, this therapeutic approach may become one of the effective strategies in alleviating the pain flares in RA patients.
